# Supplementary material for: Distribution of Wheat-Infecting Viruses and Genetic Variability of Wheat Streak Mosaic Virus and Barley Stripe Mosaic Virus in Kazakhstan
Source: Viruses. 2024 Jan 8;16(1):96. doi: 10.3390/v16010096 (PMC10819362; doi:10.3390/v16010096)

WSMV-CP-274 Primer set for WSMV — CP — 274 bp(this study)

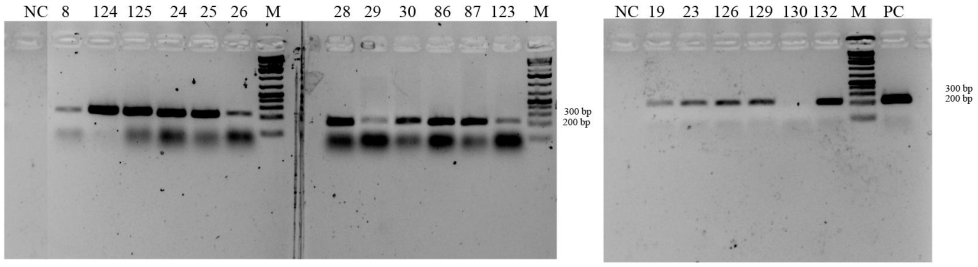

- **1.5% agarose gel**
- **M: 1 kb plus marker**
- **NC: Negative control**
- **PC: Positive control of WSMV**

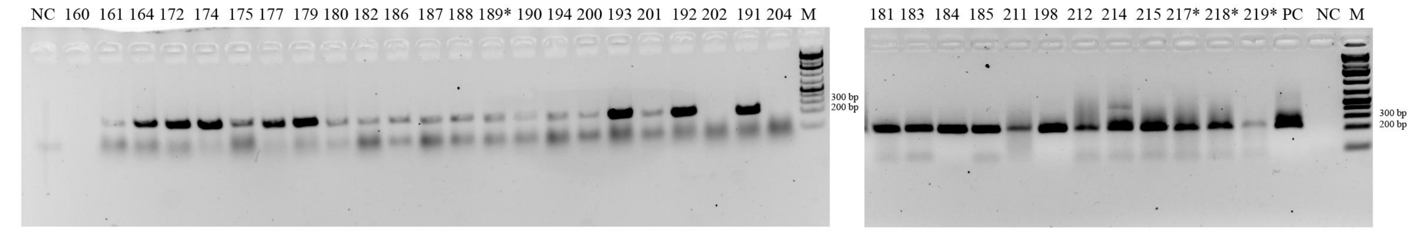

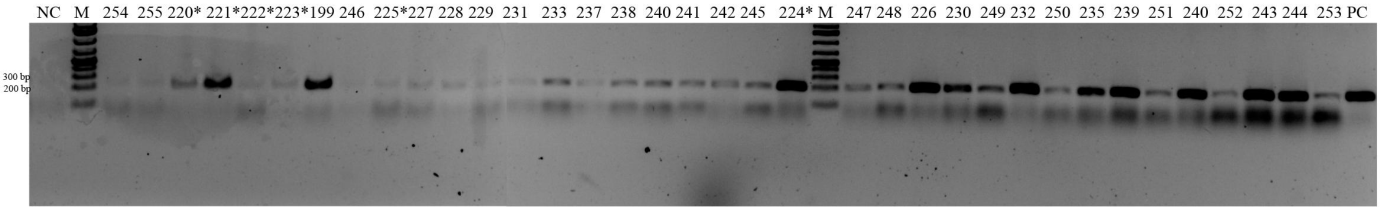

* positive samples for WSMV and BSMV

LAMP primer sets for WSMV — CP (this study)

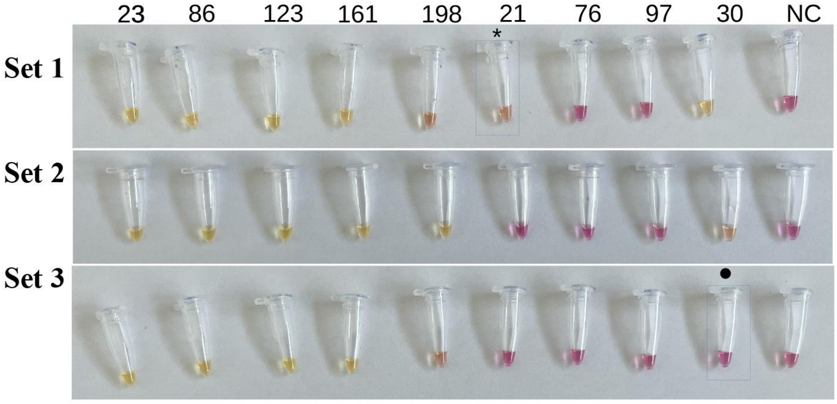

*** - False positive**

● - False negative

NC — Negative control

BSMV-γB-134 Primer set for BSMV — γB protein — 134 bp (this study)

- **1.5% agarose gel**
- **M: 1 kb plus marker**
- **NC: Negative control**
- **PC: Positive control of BSMV**

NC 3 54 73 76 37 8 59 42 21 12 36 5 21 16 38 64 56 101 110 175 203 241 256 26 M PC

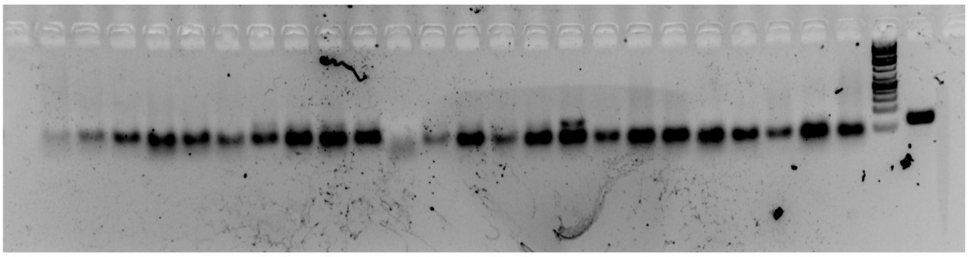

200 bp
75 bp

NC 67 81 28 72 30 91 102 13 74 105 96 77 38 189* 210 217* 218* 219* 220* 221* 48 223* 224* M 49 50 222* 68 69 82 84 88 225* PC M

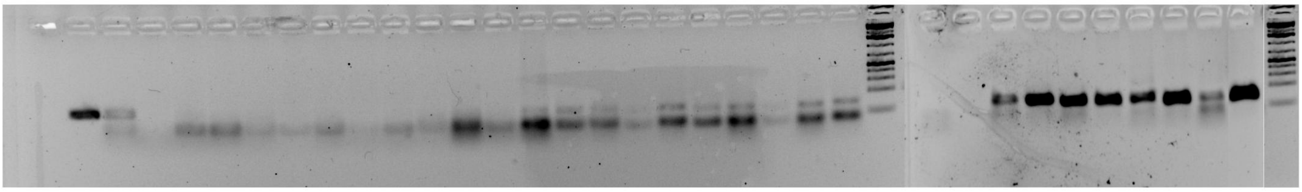

200 bp
75 bp

* positive samples for WSMV and BSMV

TGB2 Primer set for BSMV — γB protein— 397 bp (Zarzynska et al. 2014)

NC 63 64 65 67 68 69 82 83 99 100 101 102 103 M

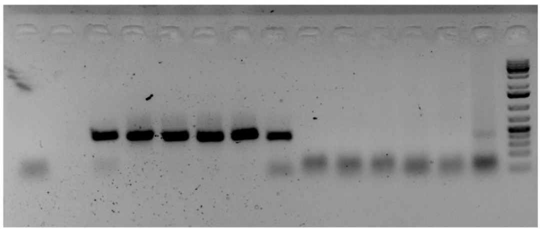

NC 203 217 218 219 220 221 222 223 224 225 226 M PC

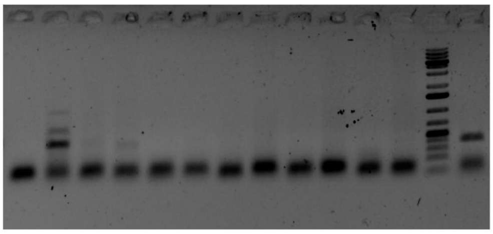

NC 84 85 86 87 M 88 89 90 91 92 93 94 95 96 97 98 PC

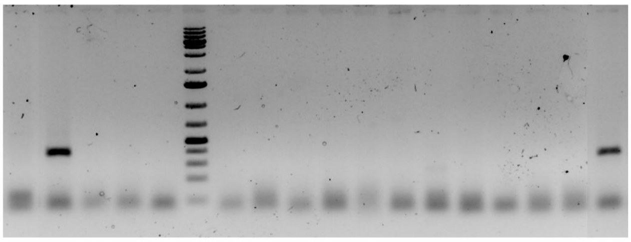

- **1.5% agarose gel**
- **M: 1 kb plus marker**
- **NC: Negative control**
- **PC: Positive control of WSMV**

WS-8166-8909 Primer set for WSMV — CP — 750 bp (Kudela et al. 2008)

NC 12 22 26 86 124 125 126 129 164 174 177 PC M

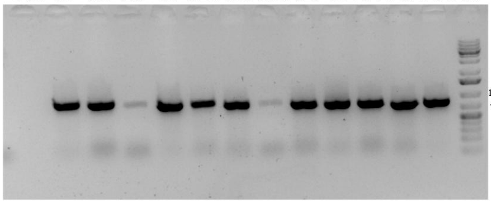

1000 bp
700 bp

NC 13 178 15 16 17 179 180 20 21 M 27 23 183 184 185 27 186 191 192 PC

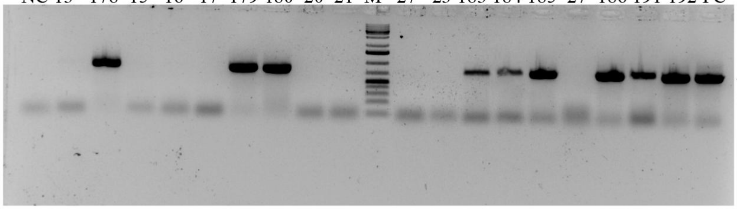

1000 bp
700 bp

NC 31 32 193 194 198 36 37 199 39 40 200 42 201 224 M PC 45 46 47 48 49 50 51 52 53 54 55 M

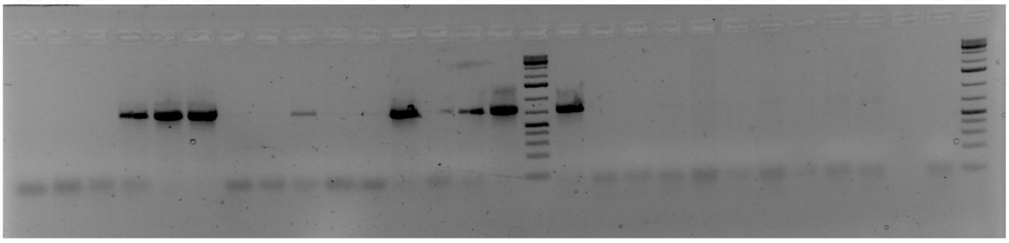

1000 bp
700 bp

- **1.5% agarose gel**
- **M: 1 kb plus marker**
- **NC: Negative control**
- **PC: Positive control of WSMV**

226 234 235 236 241 62 63 64 65 NC M 67 68 69 70 71 72 73 74 PC 75 76 77 78 M

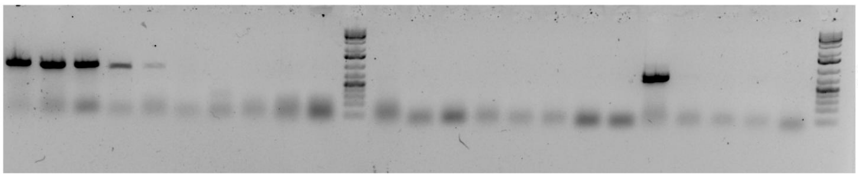

1000 bp
700 bp

WSMV Primer set for WSMV — CP — 720 bp (Byamukama et al. 2016)

86 87 88 89 90 91 92 93 94 95 96 97 98 99 M NC

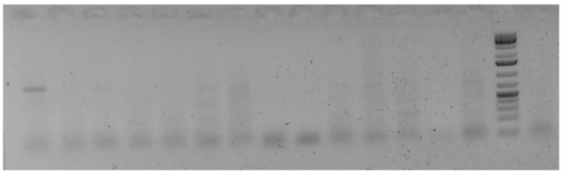

- **1.5% agarose gel**
- **M: 1 kb plus marker**
- **NC: Negative control**
- **PC: Positive control of WSMV**

NC 26 28 29 30 31 55 57 162 164 165 177 M 178 179 180 181 PC

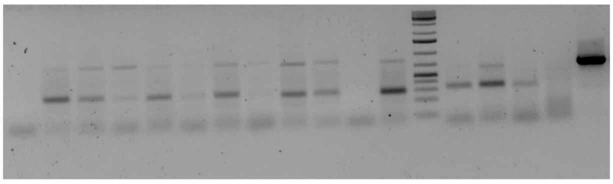

Supplement: Supplementary file 1 [file viruses-16-00096-s001.zip › Figure S1.pdf]
