# Supplementary material for: Distribution of Wheat-Infecting Viruses and Genetic Variability of Wheat Streak Mosaic Virus and Barley Stripe Mosaic Virus in Kazakhstan
Source: Viruses. 2024 Jan 8;16(1):96. doi: 10.3390/v16010096 (PMC10819362; doi:10.3390/v16010096)
Supplement: Supplementary file 1 [file viruses-16-00096-s001.zip › Table S1.pdf]

Table S1. Wheat streak mosaic virus (WSMV) and barley stripe mosaic virus (BSMV) isolates obtained from GenBank and used in sequence analysis

WSMV, BSMV * isolates	Accession number	Geographic origin	Type	Reference
KZ57	OP793649	Kazakhstan, Kostanay, Central Asia	coat protein gene	This study
KZ72	OP793650	Kazakhstan, Kostanay, Central Asia	coat protein gene	This study
KZ73	OP793651	Kazakhstan, Kostanay, Central Asia	coat protein gene	This study
KZ69	OP793652	Kazakhstan, Kostanay, Central Asia	coat protein gene	This study
KZ58	OP793653	Kazakhstan, Kostanay, Central Asia	coat protein gene	This study
KZ70	OP793654	Kazakhstan, Kostanay, Central Asia	coat protein gene	This study
KZ48	OP793655	Kazakhstan, Aktobe, Central Asia	coat protein gene	This study
KZ49	OP793656	Kazakhstan, Aktobe, Central Asia	coat protein gene	This study
KZ62	OP793657	Kazakhstan, Kostanay, Central Asia	coat protein gene	This study
KZ64	OP793658	Kazakhstan, Kostanay, Central Asia	coat protein gene	This study
KZ63	OP793659	Kazakhstan, Kostanay, Central Asia	coat protein gene	This study
KZ60	OP793660	Kazakhstan, Kostanay, Central Asia	coat protein gene	This study
KZ68	OP793661	Kazakhstan, Kostanay, Central Asia	coat protein gene	This study
KZ90	OP793663	Kazakhstan, Aktobe, Central Asia	coat protein gene	This study
KZ93	OP793664	Kazakhstan, Aktobe, Central Asia	coat protein gene	This study
KZ94	OP793665	Kazakhstan, Aktobe, Central Asia	coat protein gene	This study
KZ96	OP793666	Kazakhstan, Aktobe, Central Asia	coat protein gene	This study
KZ99	OP793667	Kazakhstan, Aktobe, Central Asia	coat protein gene	This study
KZ103	OP793668	Kazakhstan, Aktobe, Central Asia	coat protein gene	This study
KZ108	OP793669	Kazakhstan, Aktobe, Central Asia	coat protein gene	This study
KZ104	OP793670	Kazakhstan, Aktobe, Central Asia	coat protein gene	This study
KZ88	OP793671	Kazakhstan, Aktobe, Central Asia	coat protein gene	This study

KZ107	OP793672	Kazakhstan, Aktobe, Central Asia	coat protein gene	This study
KZ2 *	OP793673	Kazakhstan, Central Asia	γB protein	This study
KZ3 *	OP793674	Kazakhstan, Central Asia	γB protein	This study
KZ4 *	OP793675	Kazakhstan, Central Asia	γB protein	This study
KZ7 *	OP793676	Kazakhstan, Central Asia	γB protein	This study
KZ8 *	OP793677	Kazakhstan, Central Asia	γB protein	This study
KZ6 *	OP793678	Kazakhstan, Central Asia	γB protein	This study
Beh2	MN901877	Iran,Khuzistan, Behbahan ,Asia	coat protein gene	Masumi,M. et al.
Dezful	MN901874	Iran,Khuzistan, Dezful, Asia	coat protein gene	Masumi,M. et al.
Lolium	MN901873	Iran,Khuzistan, Andimeshk Asia	coat protein gene	Masumi,M. et al.
Beht	MN901876	Iran, Khuzistan, Behbahan Asia	coat protein gene	Masumi,M. et al.
Beh1	MN901875	Iran,Khuzistan, Behbahan Asia	coat protein gene	Masumi,M. et al.
Behbahan	MN901878	Iran, Khuzistan, Behbahan Asia	coat protein gene	Masumi,M. et al.
Chen	MN901896	Iran, Khorasan, Chenaran Asia	coat protein gene	Masumi,M. et al.
Neyshabour	MN901895	Iran,Khorasan, Neyshabour Asia	coat protein gene	Masumi,M. et al.
FASA-E	MN901897	Iran,Fars, Fasa Asia	coat protein gene	Masumi,M. et al.
Azkh132	MN901881	Iran,West-Azarbayejan, Azarshahr Asia	coat protein gene	Masumi,M. et al.
Azgh132	MN901880	Iran, West-Azarbayejan, Azarshahr Asia	coat protein gene	Masumi,M. et al.
Azsh14	MN901879	Iran,West-Azarbayejan, Azarshahr Asia	coat protein gene	Masumi,M. et al.
Miandoab	MN901882	Iran, West-Azarbayejan, Miandoab Asia	coat protein gene	Masumi,M. et al.
994	U54567	USA,Oklahoma America	coat protein gene	Sherwood,J.L. et al.
KY0074	AF511623	USA,Kentucky America	polyprotein gene	Stenger,D.C. et al.

KY00	AF511622	USA,Kentucky America	polyprotein gene	Stenger,D.C. et al.
SD96	AF511641	USA,South Dakota America	polyprotein gene	Stenger,D.C. et al.
IHC	AF511620	Canada America	polyprotein gene	Stenger,D.C. et al.
PV106JM	AF511638	USA,Ohio America	polyprotein gene	Stenger,D.C. et al.
EW95	AF511603	USA,Kansas America	polyprotein gene	Stenger,D.C. et al.
CM93	AF511600	USA,Kansas America	polyprotein gene	Stenger,D.C. et al.
H94PM	AF511610	USA,Kansas America	polyprotein gene	Stenger,D.C. et al.
OK98	AF511633	USA,Oklahoma America	polyprotein gene	Stenger,D.C. et al.
CO85	AF511601	USA,Colorado America	polyprotein gene	Stenger,D.C. et al.
MO99B	AF511628	USA,Missouri America	polyprotein gene	Stenger,D.C. et al.
MO99A	AF511627	USA,Missouri America	polyprotein gene	Stenger,D.C. et al.
GY93	AF511607	USA,Kansas America	polyprotein gene	Stenger,D.C. et al.
H95LB	AF511613	USA,Kansas America	polyprotein gene	Stenger,D.C. et al.
TX96	AF511642	USA,Texas America	polyprotein gene	Stenger,D.C. et al.
H94USDA	AF511612	USA,Kansas America	polyprotein gene	Stenger,D.C. et al.
H94S	AF511611	USA,Kansas America	polyprotein gene	Stenger,D.C. et al.
RO95	AF511640	USA,Kansas America	polyprotein gene	Stenger,D.C. et al.
ND	AF511631	USA,North Dakota America	polyprotein gene	Stenger,D.C. et al.
HM93	AF511616	USA,Kansas America	polyprotein gene	Stenger,D.C. et al.
GH95	AF511605	USA,Kansas America	polyprotein gene	Stenger,D.C. et al.

H81	AF511608	USA,Kansas<br>America	polyprotein gene	Stenger,D.C. et al.
FO93	AF511604	USA,Kansas<br>America	polyprotein gene	Stenger,D.C. et al.
CL93	AF511599	USA,Kansas<br>America	polyprotein gene	Stenger,D.C. et al.
PV91H	AF511639	USA,Kansas<br>America	polyprotein gene	Stenger,D.C. et al.
KM93	AF511621	USA,Kansas<br>America	polyprotein gene	Stenger,D.C. et al.
S81D	AF511596	USA,Nebraska<br>America	polyprotein gene	Stenger,D.C. et al.
H88	AF511609	USA,Kansas<br>America	polyprotein gene	Stenger,D.C. et al.
BT95	AF511597	USA,Kansas<br>America	polyprotein gene	Stenger,D.C. et al.
PN95	AF511636	USA,Kansas<br>America	polyprotein gene	Stenger,D.C. et al.
NE96	AF511632	USA,Nebraska<br>America	polyprotein gene	Stenger,D.C. et al.
HV91	AF511617	USA,Kansas<br>America	polyprotein gene	Stenger,D.C. et al.
LG92	AF511626	USA,Kansas<br>America	polyprotein gene	Stenger,D.C. et al.
TAMU	U54574	USA,Texas<br>America	coat protein gene	Sherwood,J.L. et al.
sidney	U54573	USA,Nebraska<br>America	coat protein gene	Sherwood,J.L. et al.
PL95	AF511635	USA,Kansas<br>America	polyprotein gene	Stenger,D.C. et al.
LC95	AF511625	USA,Kansas<br>America	polyprotein gene	Stenger,D.C. et al.
KY0083SV	AF511624	USA,Kentucky<br>America	polyprotein gene	Stenger,D.C. et al.
pv57	U53604	USA(American type culture collection)<br>America	coat protein gene	Sherwood,J.L. et al.
OSU	U54571	USA<br>America	coat protein gene	Sherwood,J.L. et al.
pv91	U54568	USA(American type culture collection)	coat protein gene	Sherwood,J.L. et al.

		America		
ATCC PV91	FJ695511	USA(American type culture collection) America	coat protein gene	Gadiou,S. et al.
ATCC PV57	FJ695512	USA(American type culture collection) America	coat protein gene	Gadiou,S. et al.
Agdia	FJ695510	USA America	coat protein gene	Gadiou,S. et al.
OSU	AF511634	USA,unknown America	polyprotein gene	Stenger,D.C. et al.
GO93	AF511606	USA,Kansas America	polyprotein gene	Stenger,D.C. et al.
CO87	AF511602	USA,Colorado America	polyprotein gene	Stenger,D.C. et al.
PV57	AF511595	USA,Kansas America	polyprotein gene	Stenger,D.C. et al.
PV106H	AF511637	USA,Ohio America	polyprotein gene	Stenger,D.C. et al.
MO00	AF511629	USA,Missouri America	polyprotein gene	Stenger,D.C. et al.
WO93	AF511644	USA,Ohio America	polyprotein gene	Stenger,D.C. et al.
CK93	AF511598	USA,Kansas America	polyprotein gene	Stenger,D.C. et al.
Washington	AF034415	USA America	polyprotein gene	Stenger,D.C. et al.
1256	KC152466	Brazil America	coat protein gene	Mar,T.B. et al.
912	KC152462	Brazil America	coat protein gene	Mar,T.B. et al.
1254	KC152465	Brazil America	coat protein gene	Mar,T.B. et al.
1233	KC152464	Brazil America	coat protein gene	Mar,T.B. et al.
915	KC152463	Brazil America	coat protein gene	Mar,T.B. et al.
486	KC020196	Brazil America	coat protein gene	Mar,T.B. et al.
964	U54570	USA,Oklahoma America	coat protein gene	Sherwood,J.L. et al.

Colorado	U54572	USA,Colorado<br>America	coat protein gene	Sherwood,J.L. et al.
Gibson	DQ888803	Australia<br>Australia	coat protein gene	Dwyer,G.I. et al.
Mt. Burdett	DQ888801	Australia<br>Australia	coat protein gene	Dwyer,G.I. et al.
Ginninderra	DQ462279	Australia<br>Australia	coat protein gene	Dwyer,G.I. et al.
SP-6	DQ462277	Australia<br>Australia	coat protein gene	Dwyer,G.I. et al.
Kondonin	DQ888805	Australia<br>Australia	coat protein gene	Dwyer,G.I. et al.
partial cds	AY858546	Australia<br>Australia	coat protein gene	Jones,R.A.C. et al.
SP-1	DQ462278	Australia<br>Australia	coat protein gene	Dwyer,G.I. et al.
SP-5	DQ462276	Australia<br>Australia	coat protein gene	Dwyer,G.I. et al.
Galong	DQ888804	Australia<br>Australia	coat protein gene	Dwyer,G.I. et al.
Yerritup	DQ888802	Australia<br>Australia	coat protein gene	Dwyer,G.I. et al.
Robatm	MN901894	Iran,Khorasan, Robatmiandasht<br>Asia	coat protein gene	Masumi,M. et al.
pp2	KY419574	Czech Republic<br>Europe	coat protein gene	Singh,K. and Kundu,J.K.
pp1	KY419573	Czech Republic<br>Europe	coat protein gene	Singh,K. and Kundu,J.K.
ar1	KY419572	Czech Republic<br>Europe	coat protein gene	Singh,K. and Kundu,J.K.
WSMV-UA-2017	MK167470	Ukraine<br>Europe	coat protein gene	Snihur,H. et al.
Jbajgah	MN901893	Iran,Fars, Bajagh<br>Asia	coat protein gene	Masumi,M. et al.
Toskana	FJ606885	Italy<br>Europe	coat protein gene	Gadiou,S. et al.
Burgund	FJ606884	Italy<br>Europe	coat protein gene	Gadiou,S. et al.

HUBA3	MT780553	Hungary<br>Europe	Polyprotein/coat protein gene	Pasztor,G. et al.
HUUS3	MT780561	Hungary<br>Europe	Polyprotein/coat protein gene	Pasztor,G. et al.
HUUS2	MT780560	Hungary<br>Europe	Polyprotein/coat protein gene	Pasztor,G. et al.
HUUS8	MT780566	Hungary<br>Europe	Polyprotein/coat protein gene	Pasztor,G. et al.
HUUS1	MT780559	Hungary<br>Europe	Polyprotein/coat protein gene	Pasztor,G. et al.
HUBA8	MT780558	Hungary<br>Europe	Polyprotein/coat protein gene	Pasztor,G. et al.
HUBA6	MT780556	Hungary<br>Europe	Polyprotein/coat protein gene	Pasztor,G. et al.
HUBA2	MT780552	Hungary<br>Europe	Polyprotein/coat protein gene	Pasztor,G. et al.
HUBA5	MT780555	Hungary<br>Europe	Polyprotein/coat protein gene	Pasztor,G. et al.
WSMVcz1	FJ216408	Czech Republic<br>Europe	polyprotein	Gadiou,S. et al.
Turkei	FJ606886	Turkey<br>Europe	coat protein gene	Gadiou,S. et al.
SK344	FJ613358	Slovakia<br>Europe	polyprotein	Gadiou,S. et al.
SlastJR	FJ216414	Czech Republic<br>Europe	polyprotein	Gadiou,S. et al.
Policko-CRI	FJ216412	Czech Republic<br>Europe	polyprotein	Gadiou,S. et al.
PoleR	FJ216410	Czech Republic<br>Europe	polyprotein	Gadiou,S. et al.
KromJR	FJ216413	Czech Republic<br>Europe	polyprotein	Gadiou,S. et al.
Podousy	FJ216411	Czech Republic<br>Europe	polyprotein	Gadiou,S. et al.
KosHJR	FJ216409	Czech Republic<br>Europe	polyprotein	Gadiou,S. et al.
SK512	FJ613359	Slovakia<br>Europe	polyprotein	Gadiou,S. et al.
HUUS6	MT780564	Hungary<br>Europe	Polyprotein/coat protein gene	Pasztor,G. et al.

HUUS7	MT780565	Hungary<br>Europe	Polyprotein/coat protein gene	Pasztor,G. et al.
HUUS4	MT780562	Hungary<br>Europe	Polyprotein/coat protein gene	Pasztor,G. et al.
HUUS9	MT780567	Hungary<br>Europe	Polyprotein/coat protein gene	Pasztor,G. et al.
HUBA4	MT780554	Hungary<br>Europe	Polyprotein/coat protein gene	Pasztor,G. et al.
HUBA7	MT780557	Hungary<br>Europe	Polyprotein/coat protein gene	Pasztor,G. et al.
HUBA1	MT260879	Hungary<br>Europe	Polyprotein/coat protein gene	Pasztor,G. et al.
Bodycek	KY419571	Czech Republic<br>Europe	coat protein gene	Singh,K. and Kundu,J.K.
Avenue	KY419570	Czech Republic<br>Europe	coat protein gene	Singh,K. and Kundu,J.K.
HUUS5	MT780563	Hungary<br>Europe	Polyprotein/coat protein gene	Pasztor,G. et al.
Hymack	KY419569	Czech Republic<br>Europe	coat protein gene	Singh,K. and Kundu,J.K.
Turondot	KY419568	Czech Republic<br>Europe	coat protein gene	Singh,K. and Kundu,J.K.
Ukraine-Ep-18	MH523357	Ukraine<br>Europe	coat protein gene	Mishchenko,L.T. and Dunich,A.A.
Ukraine-Mal-18	MH523356	Ukraine<br>Europe	coat protein gene	Mishchenko,L.T. and Dunich,A.A.
Qazvin	MN901892	Iran,Qazvin, Qazvin<br>Asia	coat protein gene	Masumi,M. et al.
Zanjan	MN901891	Iran,Zanjan, Zanjan<br>Asia	coat protein gene	Masumi,M. et al.
FASAW	MN901885	Iran,Fars, Fasa<br>Asia	coat protein gene	Masumi,M. et al.
Sedeh	MN901884	Iran,Fars, Sedeh<br>Asia	coat protein gene	Masumi,M. et al.
ShahrK	MN901883	Iran,Chaharmahal, Shahrekord<br>Asia	coat protein gene	Masumi,M. et al.
Zan132	MN901890	Iran,Zanjan, Zanjan<br>Asia	coat protein gene	Masumi,M. et al.
Kab52	MN901889	Iran,Isfahan, Kabootarabad	coat protein gene	Masumi,M. et al.

		Asia		
Isfahan	MN901886	Iran,Isfahan, Isfahan<br>Asia	coat protein gene	Masumi,M. et al.
Abargoo	MN901887	Iran,Yazd, Abargoo<br>Asia	coat protein gene	Masumi,M. et al.
Azna	MN901888	Iran, Lorestan, Azna<br>Asia	coat protein gene	Masumi,M. et al.
Qasr Ibrim*	KJ433979	Qasr Ibrim, Egypt<br>Africa	complete sequence	Smith,O. et al.
China strain*	AY787207.1	China<br>Asia	complete sequence	Sun,X. et al.
Type*	M16576.1	USA<br>America	unknown protein	Gustafson,G. et al.
Complete genome	NC_003478.1	USA<br>America	complete sequence	Gustafson,G. et al.
ND18*	M16577.1	USA<br>America	unknown protein	Gustafson,G. et al.
Complete genome	X52774.1	Argentina<br>America	unnamed protein product	Kozlov,Yu.V. et al.
XJ*	KJ746473.1	China<br>Asia	complete sequence	Yuan,C., Hu,Y. and Li,D.
Norwich*	JF803285.1	Norwich,England	complete sequence	Lee,M.Y. and Jackson,A.O.
De-M*	KY615800.1	Germany<br>Europe	complete sequence	Zarzynska-Nowak,A. et al.
R*	KY615798.1	Poland<br>Europe	complete sequence	Zarzynska-Nowak,A. et al.
M*	KY615799.1	Poland<br>Europe	complete sequence	Zarzynska-Nowak,A. et al.
M/R*	KY615806.1	Poland<br>Europe	complete sequence	Zarzynska-Nowak,A. et al.
