# Supplementary figures and images for: Distribution of Wheat-Infecting Viruses and Genetic Variability of Wheat Streak Mosaic Virus and Barley Stripe Mosaic Virus in Kazakhstan
Source: Viruses. 2024 Jan 8;16(1):96. doi: 10.3390/v16010096 (PMC10819362; doi:10.3390/v16010096)

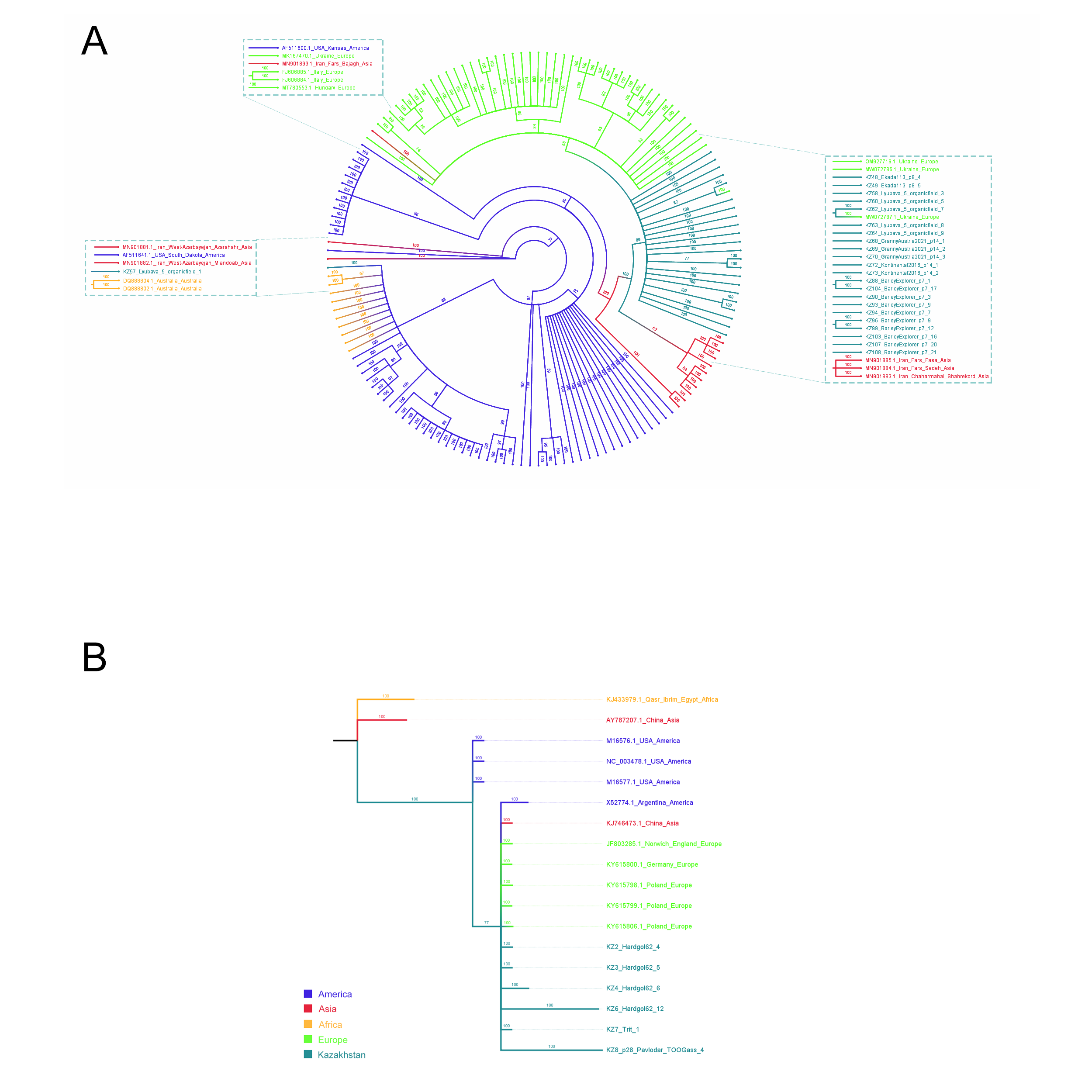

Supplement: Supplementary file 1 [file viruses-16-00096-s001.zip › figure S2.png]
